# The Southern Medical and Gynecological Association

**Published:** 1895-12

**Authors:** 


### THE SOUTHERN MEDICAL AND GYNECOLOGICAL ASSOCIATION.

The eighth annual meeting of this association was held on November 12th, 13th and 14th, in Washington, D. C., under the presidency of Dr. L. McLane Tiffany of Baltimore.

The various sections of the North and South were well represented, and many important papers were read and discussed, though a number of those whose names were on the program were absent, and their papers were only read by title. The following were presented: "Personal Experience in the Treatment of Stab Wounds of the Intestines and Peritoneum," by Bedford Brown, M.D., Alexandria, Va.; "Report of Five Cases in Abdominal Surgery in Which the Murphy Button Was Applied," by A. Vanderveer, M.D., Albany, N. Y.; "Resections and Intestinal Anastomosis," by H. Horace Grant, M.D., Louisville, Ky.; "Necrosis of the Ribs in Three Cases, Resultant in Typhoid Fever," by W. M. Robinson, M.D., Danville, Va.; "Surgical Interference in Rectal Disorders," by J. McFadden Gaston, M.D., Atlanta, Ga.; "Cancer of the Pregnant Uterus," by Geo. H. Noble, M.D., Atlanta, Ga.; "Hysterectomy for Fibroids," by E. S. Lewis, M.D., New Orleans, La.; "The Technique of Supravaginal Hysterectomy," by Howard A. Kelly, M.D., Baltimore, Md.; "Some of the Reasons for Preferring Vaginal to Abdominal Section for Pain in the Pelvis," by Joseph Tabor Johnson, M.D., Washington, D. C.; "Indications and Technique of Vaginal Hysterectomy," by Geo. H. Rohe, M.D., Cantonsville, Md.; "Hysterectomy for Puerperal Sepsis," by A. M. Cartledge, M.D., Louisville, Ky.; "Management of Cases Which Have Recovered from Appendicitic Abscess, in Which the Appendix Was not Removed," by J. D. S. Davis, M.D., Birmingham, Ala.; "The Comparative Frequency of Stones in the White and Negro Races," by George Ben Johnson, M.D., Richmond, Va.; "Report of Cases of Cystotomy for Stone, and of Tracheotomy for Foreign Bodies in the Trachea," by W. F. Westmoreland, M.D., Atlanta, Ga.; "Annual Address of the President," by L. McLane Tiffany, Baltimore, Md.; "Dr. J. Marion Sims and His Work," by John A. Wyeth, M.D., New York; "The Management of Intestinal Complaints in Intra-pelvic Operations," by L. S. McMurtry, M.D., Louisville, Ky.; "Abdominal Pregnancy," by Cornelius Kollock, M.D., Cheraw, S. C.; "Report of a Case of Extra-uterine Pregnancy," by John T. Murry, M.D., Chester, S. C.; "The Technique of the Buried Suture," by Henry O. Marcy, M.D., Boston, Mass.

Out of thirty-seven papers on the program, twenty-one were read, and there was a thorough discussion of a large number, rendering the meeting very interesting and instructive to all who were present. Short addresses, in connection with the tributes to Dr. Sims, were read by Drs. Engelman, Nelson, Kollock, Vanderveer, Wilson, Marcy, Gaston, Westmoreland, and Robinson. A gold-mounted gavel, made from the operating-table of Dr. J. Marion Sims, was presented by his son, Dr. H. Marion Sims, of New York.

The next place of meeting for the association is at Nashville, on the second Tuesday of November, 1896.

The officers elected for the ensuing year are as follows :

For President—Dr. E. S. Sims, New Orleans, La.

Vice-President—Dr. J. Tabor Johnson, Washington, D. C.

Vice-President—Dr. Richard Douglas, Nashville, Tenn.

Secretary—Dr. W. E. B. Davis, Birmingham, Ala.

Treasurer—Dr. A. M. Cartledge, Louisville, Ky.

This meeting of the Southern Surgical and Gynecological Association was rendered pleasant by the reception extended from the Obstetric and Gynecological Society, and by Dr. J. Tabor Johnson, on the evenings of the first and second days. J. M'F. G.

---

DR. J. EMMETT BLACKSHEAR died in Savannah November 15th, aged sixty-five. Dr. Blackshear had practiced medicine in Marietta, Macon, Atlanta, and Savannah. In Macon he was for a number of years city physician and president of the board of health. At the time of his death he was grand secretary of the Grand Lodge of Masons of Georgia. He was buried in Macon.

---

DR. M. P. DEADWYLER, of Elberton, died November 5th. Dr. Deadwyler was a highly esteemed physician, and for thirty years had enjoyed a large practice in his community. He has been a member of the Georgia Medical Association since 1868.

